# Supplementary material for: A network model of affective odor perception
Source: PLoS One. 2020 Jul 30;15(7):e0236468. doi: 10.1371/journal.pone.0236468 (PMC7392242; doi:10.1371/journal.pone.0236468)
Supplement: Attachment — Submitted filename: Reply_to_reviewers_NOFC.docx [file pone.0236468.s002.docx]

| Q | Comments of Reviewer #2 | Author’s reply |
| --- | --- | --- |
| 1 | First of all, the tool they use to measure mood is not suitable for fine mood measurement. The authors should better justify the use of EmojiGrid to characterize the mood of participants. It is obvious that positioning one's mood on a two-dimensional space of valence and arousal allows to obtain global information on this mood. But this same space reduces the possibility to dissociate qualitatively different mood states. This is why standardized mood questionnaires such as POMS (Profile of Mood States) exist, which contains more than two valence and arousal scales. Using the EmojiGrid, how to dissociate a state of anxiety (negative valence and high arousal) from an irritable state (negative valence and high arousal). I think you would agree that these moods are not identical and could influence olfactory perception in a specific way. This is problematic because the authors aim to make the link between mood, olfactory perception and olfactory emotional response. How can they answer this question if they do not measure mood according to scales that capture best the quantitative and qualitative differences in mood? See for instance: “Profile of Mood States.” McNair, D. M., Lorr, M., & Droppleman, L. F. (1992). Revised Manual for the Profile of Mood States. San Diego, CA: Educational and Industrial Testing Services., no. 65 (37), | We agree with the reviewer that (1) different moods can influence olfactory perception in different ways, and (2) the EmojiGrid does not characterize all aspects of mood. However, we did not intend to measure the effect of all possible mood states on the affective appraisal of odors. In this study we focused on the valence and arousal components of the baseline (core) affective state (using the EmojiGrid), since these might influence the valence and arousal ratings subsequently obtained for the different odors. To clarify this issue, we replaced “mood” by “valence and arousal components of affective state”, and we emphasize this restriction in the limitations section. |
| 2 | Second, using the detection threshold of a single molecule in the model is problematic. The authors determine the AEP detection threshold for each participant and use this value to establish the influence of the threshold on odour-related emotional response. This could have been a very important contribution if the authors had performed the detection threshold for each odour used in the affective assessment (I can absolutely not blame the authors for not doing it because it would be an extremely tedious job). Thresholds are specific to each molecule; determining a perception threshold for one molecule does not predict thresholds for others. The test used for the threshold is effective in clinical investigation to detect possible anosmia or hyposmia. This allows the patient to be quickly located according to a known standard. But the approach of the authors in this research is to observe systematic relations between detection thresholds, perceptive and affective dimensions thanks to the use of 40 different odorants. It is not by introducing the threshold to one and only one molecule that these systematic relationships can be studied. One argument for my criticism is that the network analysis the authors performed does not reveal a relationship between threshold and perceived intensity. If the threshold measurement with one molecule was a good estimate of overall olfactory capacity, then a strong positive relationship would have been expected. Hsieh, J. W., Keller, A., Wong, M., Jiang, R. S., & Vosshall, L. B. (2017). SMELL-S and SMELL-R: olfactory tests not influenced by odor-specific insensitivity or prior olfactory experience. Proceedings of the National Academy of Sciences, 114(43), 11275-11284. | We thank the reviewer for pointing out this issue.  In this study we measured olfactory sensitivity using the validated Sniffin’ Sticks based threshold test (SST), which has become a popular (validated and standard) procedure in the literature. This test was also used in several references cited in this study, allowing us to relate our current finding to those earlier results. We realize that the SST test is indeed a single-molecule based general diagnostic tool to assess olfactory functioning, which cannot distinguish between general smell dysfunction and PEA insensitivity Hsieh et al., 2017. We agree that it would be more informative to measure detection thresholds for each of the 40 odors individually, and that this might have resulted in a stronger positive relationship between threshold and intensity. However, such a procedure would indeed have been too time consuming and tiresome for the participants.  Future studies might consider the use of a threshold test based on complex odor mixtures to obtain olfactory threshold assessments that are less dependent on the individual variability in sensitivity to specific odorants (e.g. SMELL-R: Hsieh et al., 2017; see also Oleszkiewicz et al., 2017). However, this only appears to hold for specific odor mixtures Oleszkiewicz et al., 2018, and there is currently no generally accepted and validated complex-odor based threshold test available.  We now address these issues in the Limitations section. |
|  | Introduction |  |
| 3 | Line 27: Just because there is a high degree of overlap between the cerebral systems that process olfaction and emotions does not mean that the responses have to be "various", this overlap could exist with only one emotional response. Please rephrase or delete that attempted explanation. | We rephrased the text as follows: “Odors can effectively elicit affective responses” |
| 4 | Line 30: These dimensions are not necessarily the most fundamental, they are fundamental but not the only ones. They are especially the most used to measure the "core affect". Fontaine, J. R., Scherer, K. R., Roesch, E. B., & Ellsworth, P. C. (2007). The world of emotions is not two-dimensional. Psychological science, 18(12), 1050-1057. | We agree with the reviewer, and changed the text accordingly: “Arousal and valence (pleasantness or hedonic tone) are the most widely used principal dimensions of affective responses to environmental stimuli in general” |
| 5 | Line 32 and following: I don't understand why the authors use the term "determined" in this sentence, it suggests that there is the valence and arousal of smell (are these intrinsic properties of smell?) that cause the emotional response. Valence and arousal are dimensions of the affective response. The authors may mean that it is possible to describe or characterize the affective response through the psychological dimensions of valence and arousal. There is abundant literature that describes the emotional response as multi-componential, with its subjective, physiological, motivational, cognitive and expressive dimensions. The emotional response cannot be limited to the subjective dimensions of valence and arousal. I suggest that the authors reduce their rationale to the characterization of the relationships between the subjective dimensions of olfactory evaluations. | We agree with the reviewer and reduced our rationale to the characterization of the relationships between the subjective dimensions of olfactory evaluations. |
| 6 | It seems that the authors differentiate between the intensity of odour, which would be a subjective quality and which they name “perceived intensity”, while they might suggest that the other dimensions represent intrinsic properties of odour, which of course are not. I think it is important to clarify that from the moment that participants are asked to evaluate odour on any dimension, this dimension is subjective and deserves the qualifier "perceived". Thus, the authors could remove the mention of “perceived” in “perceived intensity” or, on the contrary, add this mention to the other dimensions. | We replaced “perceived intensity” with “intensity” throughout the text. |
|  | data availability and analysis strategy |  |
| 7 | 1. All edges should be undirected except those from Mood which should go from mood to the other variables. Authors can’t postulate mutual relationship in this case because they don’t measure the effect of each odor on participant’s mood. | This is indeed correct: the initial or baseline affective state is of course not affected by subsequent odor presentations. To clarify this the edges connected to BV and BA in the graphs in Fig 1 and Fig 5 are now directed.  The fact that these edges are directed was explained at the end of the Introduction in section 4. |
| 8 | 2. Authors used Gaussian graphical model but didn’t give evidence there data continuous data are distributed. By deliberately selecting a group of positive and a group of negative odours, I fear that the authors have favoured non-normal valence distributions. | As mentioned in Section 2.5.2, a nonparanormal transformation was applied to Gaussianize the input before estimating the GGM. We used the nonparanormal transformation *huge.npn* from the *huge* R package Zhao et al., 2012 to normalize the data (see e.g. Epskamp & Fried, 2018;Liu et al., 2009 |
| 9 | 3. It seems to me that the measurements are repeated in the same individual (all smells are judged by each individual). Why didn't the authors take this factor into account when applying the GGM? In the model presented by the authors, it seems to me that each observation is considered unique and independent (cross-sectional data analysis) which is not the case. | Thank you for pointing this out: Each participant did indeed judge each odor. To attenuate interindividual variance (as this is not the main interest of this paper) while retaining within-subject variance, we re-analyzed the data, this time after converting the valence, arousal, intensity and familiarity scores per individual and per odor valence set (pleasant/unpleasant) to z scores. This new analysis retains the overall structure of the main relations in both networks as reported in the previous draft version, while eliminating some spurious relations. As a result, the accuracy and stability of the networks has improved. |
| 10 | 4. I particularly appreciate the willingness of the authors to share the data from this experiment along with the analysis scripts. Indeed, this is a practice that must now be encouraged if science is to evolve in complete transparency. However, in the data shared by the authors, participant numbers and odours’ names are not reported, which limits the verification and exploitation of the data. I strongly encourage the authors to provide this information which will be very important for the olfactory research community. | We now added an Excel file to the Supplementary Material, reporting all raw data, including the participant identifiers and odour names, as well as the means, SD and other ratings for all odorants (see Q 20). |
|  | Discussion |  |
| 11 | The authors conducted the analyses separately for positive and negative odours to “avoid neutralization effects, and because of the functional dichotomy that may exist in the relation between valence and the other variables that are measured in this study (e.g., the relation between valence and familiarity: [37])” The results they present suggest that pleasant and unpleasant networks do not differ. What are the functional consequences of this result? What does this result tell us about the nature of the relationships between the subjective odour dimensions or the treatment of pleasant and unpleasant odours? There are still some small differences between the two networks but it is very difficult for the reader to imagine the importance of these differences. | In spite of the fact that pleasant and unpleasant odors are processed in different brain regions, we find that the relation between their affective appraisal (valence, arousal) and the other variables that are measured in this study (e.g., familiarity, intensity) is highly similar. This suggests that the affective appraisal of odors (and probably also of signals from other sensory modalities) is mediated by a general framework that is independent of the exact nature of the underlying brain regions that initially processed the odor. We now address this in the discussion. |
|  | Comments of Reviewer #3 | Author’s reply |
| 12 | Although I feel a model of affective odor appraisal should probably include additional contextual factors going beyond mood; mood can be considered context, and so this is an important step towards an integral model on affective odor appraisal. The authors do point out that future models should include gender and personality, for example. The authors should acknowledge that this may change the relations in the model quite a bit. For example, risk perception from odor exposure or source of exposure will affect the relations. | We now discuss these issues in the section on Limitations. |
|  | Introduction |  |
| 13 | I am missing a bit of nuance in certain paragraphs like 1.2.1 on perceived intensity. For example, the suggested relation between detection thresholds, intensity and valence should receive more attention. One could interpret the paragraph as implying that individuals with high thresholds show lower intensity perception or different valence perception. In view of the importance of perceived intensity in this paper, please extend this paragraph to include a section on threshold, determinants of perceived intensity and their relation. | In section 1.2. we added a discussion on the relation between detection threshold and some of the determinants of perceived intensity. |
| 14 | There is more attention going to familiarity in 1.2.2, but what I miss is a clear definition of familiarity and how it relates to recognition/identification because it is my impression these terms are mixed up later on in the paper. I would recommend the authors refer to Kösters misfit theory. | We added a definition of familiarity: “the feeling that an odor is known or has been perceived before”. Please note that we did not investigate odor recognition or identification in this study: “The participants were explicitly asked not to attempt to identify the smells” (section 2.4 Procedure).  We also discuss how familiarity relates to affective odor appraisal and recognition/identification:  “Familiarity is implicitly linked to the affective and hedonic appreciation of our environment rather than to explicit source recognition Köster et al., 2014. Olfaction appears to serve novelty or change detection (possibly mediated by the amygdala: Blackford et al., 2010;Krusemark et al., 2013;Nigri et al., 2013), directing our attention to odors that are either unknown (not experienced before: categorical novelty) or do not fit our expectation or previous experience of a given situation (contextual novelty or misfit; see Köster et al., 2014).” |
| 15 | 1.3 Valence and arousal What is not addressed here (unless I missed something) is the importance of learning. The general notion in the (human) olfaction literature is that odor valence, with perhaps very few exceptions, is the result of learning, rather than “innate” or “labelled line”. So the context in which an odor was experienced will determine how that odor is liked. While for many individuals context may be similar (e.g. delicious foods) resulting in same direction of valence, the resulting valence may vary (considerably) between individuals. It is important to address this in the paper, to set the stage for the modelling. This is to complete the set of factors we would ideally deal with. | We added a discussion on the relevance of learning:  “Note that the valence of odors may change due to learning effects. While affective odors appraisal appears to be partly innate Khan et al., 2007;Mandairon et al., 2009;Soussignan et al., 1997, factors like the frequency and context of prior exposure, semantic knowledge, and cultural background can cause significant variations in hedonic perception between individuals and over the course of the human life-span Rouby et al., 2009. For instance, odors that are initially perceived as neutral or positive may acquire a negative connotation and signal threat after they have been experienced in the context of negative life events Li, 2014.” |
|  | Methods |  |
| 16 | I am not an expert on network analysis. Since the authors themselves reflect on sample size and (lack of) statistical power in the Discussion, can they reflect on whether the sample size of 56 for this type of analysis? How does this reflect on model fit and related parameters? Have they done a power analysis? I realize testing participants on odor perception in the lab requires substantial effort. | We now address this issue in section 2.1 Participants.  To conduct a power analysis (determine the adequate sample size) an expectation of the effect size is required. The network equivalent is an expected (weighted) network structure (Epskamp et al., 2018). However, since this is the first study of this type, no previous similar networks were available. Sample size was therefore determined from a general rule of thumb suggested in the literature; namely, three individuals per parameter (Epskamp et al., 2018). Since the HOEA network has 14 edges, this means that the study needed a minimal group size of 42 participants to meet this “rule of thumb”. Our sample size of 56 was also considered adequate given the outcomes of the bootstrap accuracy and stability analysis reported in section 3.2.2. |
| 17 | Iso-intensity Testing for iso-intensity of the stimulus set requires more than three authors sniffing the stimuli to agree on intensity. Typically, this would require preparing multiple concentrations of the stimuli, having panelists smell and rate them and titrate the concentrations to get to iso-intensity over multiple sessions. Can the authors elaborate on what they did to achieve iso-intensity? However, I am confused as to the need for iso-intensity. Intensity is a factor in the model and therefore it needs to vary. Judging from the outcome, it does, which would contradict the earlier statement of iso-intensity. Why would you need equal intensity in the first place? Perhaps the authors can clarify this. | Please note that we did not aim to achieve iso-intensity, we merely wanted the intensities of the different odors to be comparable (i.e., not too extremely different), since large differences in intensity may confound the affective ratings because of the inverse valence-intensity relation. |
| 18 | Table 1: Please include concentrations (% compound in diluent or otherwise) in the Table for replication purposes. What did the safety assessment of compounds like tar, turpentine, and diesel fumes entail? | The composition of the stimuli is explained in section 2.2: Stimuli. The RS codes are given in Table I. The specific concentration values are not provided by the suppliers. We refer to the commercial suppliers (www.burghart-mt.de, retroscent.com, www.hekserij.nl) for their specific safety assessment protocols. |
| 19 | Procedure (page 14): - It is always advised to have repetitions on all odor ratings. However, unless I missed this, I guess with 40 odors this was not possible. | This is correct: each of the 40 odors was only presented once to each participant. |
|  | Results |  |
| 20 | - Please provide a Table with means, SD or thresholds and other ratings for all odorants | We added an Excel file reporting all raw data with participant identifiers and odour names, as well as the means, SD and other ratings for all odorants (see also Q 10). |
| 21 | - How are individuals who do not judge peach as pleasant, perceive pineapple as lowly intense and peppermint as not familiar, outliers? There is no wrong or right answer as to how pleasant peach should be. See my previous comment about learning. These responses are not “erroneous” (page 20) so please remove. Outlier exclusion is only acceptable if a standard from exclusion is given based on e.g. 2-3 SD’s, so if there is a statistical reason as you model would be radically different perhaps if you included those. | We explained how outliers were identified in Section 2.5.1 General statistics: “To identify outliers, we transformed all ratings for each variable and for each odor into standardized values in SPSS. Participants with standardized values exceeding two standard deviations from the mean were considered as outliers.”  In the Results section we merely provided additional information on the nature of the outliers. |
| 22 | - I am surprised by the finding that mood valence correlates strongly and negatively with threshold. I do not recall having ever seen this relation between lower threshold and positive mood before, even when the authors cite reference 60. I feel it would be important to test the model on another data set of different individuals/odors, so on new data. Will these relations hold? It would have been stronger if separate data sets had been used for model development and testing. But perhaps this is not a common approach for this type of data-driven analysis? Can the authors address this in the Discussion? | We found that the valence component of current affective state correlates strongly and negatively with the odor detection threshold (R12), both for pleasant and unpleasant odors. This result agrees with the finding that negative mental states raise odor detection thresholds (Flohr et al., 2017), while a positive mental state can lower detection thresholds (Pollatos et al., 2007). We agree with the reviewer that this result is somewhat surprising, since (to the best of our knowledge) the finding that mood valence correlates strongly and negatively with threshold has not been explicitly reported in the literature. Future studies are needed to investigate whether this relation can be replicated with different odors and populations. We now address this issue in the text. |
| 23 | Finally, it is “Utrecht University” and not “University of Utrecht” | Corrected |
