# Supplementary material for: A network model of affective odor perception
Source: PLoS One. 2020 Jul 30;15(7):e0236468. doi: 10.1371/journal.pone.0236468 (PMC7392242; doi:10.1371/journal.pone.0236468)
Supplement: Attachment — Submitted filename: Reply_to_reviewers.docx [file pone.0236468.s003.docx]

| Comments of Reviewer #2 | Author’s reply |
| --- | --- |
| Since the hypothetic model is based on the literature and the model obtained in this study on experimental data related to a sampling of specific odours and participants, how can a meaningful comparison be made? Are the differences not explicable only in terms of sampling?  Moreover, the hypothetical model proposed by the authors does not separate pleasant from unpleasant odours (it is crucial for some relations like R3). This makes any comparison very difficult. Why not just removing the theoretical model just report the existing relationships. Or the theoretical model must be separated according to the valence of the odours. | The theoretical model only serves as a reference framework, showing how the different factors that have been shown to contribute to the affective perception of odors are related, and making it easier to refer to these different relations in the rest of the paper. We agree that it was confusing to condense the results from the literature for positive and negative odors into a single graph. Therefore, we now distinguish two hypothetical relational models based on the literature: one for pleasant and one for unpleasant odors (see Figure 1). The relations R3 (lacking in the UPOEA model) and R14 (opposite sign in both models) differ between both models. |
| The analyses performed are based on linear correlations between the variables. Don't the authors think that this forced them to exclude any relation of another order (quadratic etc..) and therefore: i) to have to separate pleasant from unpleasant odours and ii) to conclude that there are two different treatments for the different valences. In other words, does the conclusion that pleasant and unpleasant odours must have specific treatments not simply follow from the fact that the analytical techniques employed examine linear relationships? I think the authors should discuss this point (linearity)a little bit more within the limitations. | We now address this issue in the Limitations section: “Using Gaussian graphical models, we implicitly assumed that the variables in our models are linearly related. Diagnostic scatterplots show that this assumption is met within the groups of pleasant and unpleasant odors. Although the use of a different kind of correlation estimate (e.g., Spearman of Kendall) would allow for modelling non-linear relations (such as the one shown in Figure 4) these models would be less optimal and new methods are needed to construct a fitting network model.”  Please note that we decided up front to analyze unpleasant and pleasant odors separately, given the evidence from the literature for the existence of different processing channels (Section 1). |
| Line 30 to 36: The three sentences introduce the same notion. Consider rewording them to make the information less redundant. | We reformulated these three sentences to reduce this redundancy. |
| Line 43 and following: I don't quite understand the authors' inference that "These physiological parameters are linked to arousal..." It is a fairly robust finding in the psychophysiological literature that electrodermal activity is sensitive to arousal but that cardiac variations can be related to valence. An inverted U-curve between the skin conductance responses amplitude and the valence is very often observed for smells but also for images and sounds. This is interpreted as a reflection that subjective arousal and subjective valence are not independent. Alaoui-Ismaili, O., Robin, O., Rada, H., Dittmar, A., & Vernet-Maury, E. (1997). Basic emotions evoked by odorants: Comparison between autonomic responses and self-evaluation. Physiology and Behavior, 62, 713–720. Alaoui-Ismaili, O., Vernet-Maury, E., Dittmar, A., Delhomme, G., & Chanel, J. (1997). Odor hedonics: Connection with emotional response estimated by autonomic parameters. Chemical Senses, 22, 237–248. Bensafi, M., Rouby, C., Farget, V., Bertrand, B., Vigouroux, M., & Holley, A. (2002a). Autonomic nervous system responses to odors: The role of pleasantness and arousal. Chemical Senses, 27, 703–709. Bensafi, M., Rouby, C., Farget, V., Bertrand, B., Vigouroux, M., & Holley, A. (2002b). Influence of affective and cognitive judgments on autonomic parameters during inhalation of pleasant and unpleasant odors in humans. Neuroscience Letters, 319, 162–166. Delplanque, S., Grandjean, D., Chrea, C.,Coppin, G.,Aymard, L.,Cayeux, I.,etal. (2009).Sequential unfolding of novelty and pleasantness appraisals of odors: Evidence from facial electromyography and autonomic reactions. Emotion 9, 316–328.doi:10.1037/a0015369 Lang, P. J., Greenwald, M. K., Bradley, M. M., & Hamm, A. O. (1993). Looking at pictures: Affective, facial, visceral, and behavioral reactions. Psychophysiology, 30, 261–273. Pichon, A., et al. Sensitivity of Physiological Emotional Measures to Odors Depends on the Product and the Pleasantness Ranges Used. In: Frontiers in Psychology, 2015, vol. 6. doi: 10.3389/fpsyg.2015.01821 | We agree with the reviewer and reformulated this part of the text. |
| Line 56 and following: I confess to having some difficulty with the use of the term "influence" in sentence propositions such as "Factors influencing the affective appraisal of odors". This type of sentence implies a causal relationship between factors that would be primary and the affective appraisal of odour that would depend on these factors. I don't think it is possible to postulate this kind of causal relationship, and the experiment conducted by the authors and the partial correlation analyses do not allow us to infer a causal link. At best, the authors can examine the variables that covariate or correlate with the affective appraisal. Is an odour judged more intense then another because it is appraised as more unpleasant? Or is it the contrary? | We agree with the reviewer that the term “influence" implies a causal relation that cannot be postulated. We therefore replaced the term “influence” by “related to” |
| Line 102: The sentence does not give a clear definition of familiarity.  I also have difficulty understanding what the authors mean when they write: “Familiarity is implicitly linked to the affective and hedonic appreciation”. Initially, the authors seem to differentiate between affective and hedonic, but there is no mention of differences in the manuscript. Is it necessary to make this distinction? Second, can familiarity be defined without resorting to the hedonic value? Here is a definition that the authors can put in the context of olfaction: “A form of remembering in which a situation, event, place, person, or the like provokes a subjective feeling of recognition and is therefore believed to be in memory, although it is not specifically recalled.” | In our previous revision we included a definition of familiarity in the first line of section 1.2: “odor familiarity (the feeling that an odor is known or has been perceived before: [33])”  This definition appears to be similar to the one that is kindly suggested here.  We removed the term “hedonic” since it is confusing and there is indeed no need to make a distinction between affective and hedonic, or to include this term in the definition of familiarity. |
| Line 114: “This suggest that pleasant and unpleasant odors are evaluated by different processing channels, in agreement with models that suggest that different evaluative channels are involved in the processing of negatively and positively valenced stimuli” This sentence doesn't add much with the two parts together. Please consider removing the first part. | We agree and removed the first part of this sentence. |
| Line 279: is it an arithmetic or geometric mean? | We replaced the term “mean” by “arithmetic mean” |
| Line 388: is the parameter λ the sparsity parameter? Is so please mention it. | We now explain that the parameter λ controls the level of sparsity |
| Line 419: Please check/correct the definition of betweenness. | We rephrased this definition as follows: “which quantifies how often one node lies on the shortest path between two other nodes” |
| Line 536, Table 2: Looking more closely (thanks to the excel file the authors provided) at the correlation between valence and familiarity for unpleasant odours only, conventional correlational analyses give no statistically significant relationship but the correlation coefficient is negative. This result is consistent with several studies in the literature cited by the authors failing to demonstrate any relationship between familiarity and valence for unpleasant odors. How then can we explain that a significant positive relationship appears in network analyses (R3, UPOEA) for the very same odors? | This discrepancy occurs because the relation R3 is not ergodic, so that the generalization from the group level to the individual level cannot be made.    The correlation between the mean (over all participants, i.e. at the aggregate or group level) valence and familiarity values provided in the Excel sheet is indeed negative (-0.38). However, at the individual level this correlation is positive.  In this paper, we use individual standardizing, i.e., the data for each individual is standardized before the network approach to retain a within-individual approach to the data.  The (seemingly counterintuitive) difference between correlation at the group and at the individual level can for instance be illustrated by the relationship between typing speed and the percentage of typos made (Hamaker, 2012). At the population level, typing speed and typos made will be negatively related. This is because as skill level increases, so does typing speed. Therefore, the faster people can type, the less likely they are to make a typo (since they are more experienced). However, examining this same relationship on a within-person level produces the opposite relationship: as typing speed increases, the number of typos will also increase, yielding a positive correlation. This makes sense because if an individual was to type faster and faster, (s)he would continue to make more mistakes.  Hamaker, E.L. (2012). Why researchers should think "within-person": A paradigmatic rationale. *Handbook of research methods for studying daily life.* pp. 43-61. New York, NY, US: The Guilford Press.  We now address this in the Discussion section:  “In contrast to previous studies that reported no consistent relation between familiarity and valence (R3) for unpleasant odors Ayabe-Kanamura et al., 1998, Delplanque et al., 2008, Ferdenzi et al., 2013, we find that R3 is positive, independent of odor valence. This discrepancy most likely arises from the fact that these earlier studies computed the correlations at the group level, while we use individual standardizing (i.e., the data for each individual is standardized before computing the networks, to retain a within-individual approach to the data).Thus, it appears that at the individual level, increasing familiarity (i.e., a reduction of uncertainty) consistently enhances valence, both for pleasant odors (valence becomes more positive) and for unpleasant ones (valence becomes less negative). This agrees with the general tendency to attribute more weight to affective information in conditions of uncertainty Faraji-Rad & Pham, 2017. Unpleasant odors that are unfamiliar (i.e., for which it has not yet been established whether they are harmful) may be rated as more unpleasant than unpleasant odors that are more familiar and known to be harmless. This also seems to agree with the finding that the unpleasant odors of fish and garlic were rated as less unpleasant by children who correctly identified them Martinec Nováková et al., 2015.” |
| Line 548: typo () -> ) | corrected |
| Line 557: For pleasant smells, familiarity and valence differ only in the strength parameter. How do we know if this is different enough to conclude that familiarity governs positive odour ratings? Is the answer at line 612 (“we cannot confidently conclude that any node in the PUOEA network is significantly more central than any other”)? | We agree with the reviewer that this conclusion cannot be made, and we therefore adjusted the text as follows: “F scores highest on all three centrality indices in the POEA network, indicating that this factor significantly contributes to the affective appraisal of pleasant odors. “ |
| Line 597 and following: I have some difficulty understanding the authors' reasoning. Do they mean that the sample mean being included in the CI of the bootstrap mean, then the accuracy is good? And then because positive and negative edge so not share CI, they are clearly different? Please reformulate if possible. | We clarified this issue in the text: “This figure shows that the confidence intervals of positive and negative edges do not overlap, meaning that edges with opposite signs in Figure 5 are significantly different. However, the edge weights of some positive edges in Figure 5 (e.g., R2, R5, R14) may not be significantly different on the population level, since their confidence intervals show a large degree of overlap” |
| Line 691: please add a nuance like “as measured in term of valence and arousal” because there are other emotion theories (not only dimensional models) and you could have chosen to assess affective appraisal via rating on basic emotions. | We agree and added the nuancing text suggested by the reviewer |
| Line 718: The authors should not link the networks they highlight at the level of the subjective variables measured to any brain functioning. The structure of the networks they obtain can in no way reflect any brain functioning. Their conclusions should be limited to associative networks only. | We removed all references to brain functions. |
| Line 744 and following: I thought partial correlations can be indicative of potential causal pathways. As mentioned by Epskamp and Fried, 2018): “…partial correlation networks are thought of as highly exploratory hypothesis-generating structures, indicative of potential causal effects.” I think that the authors have to take precautions with the notion of causality. Even if it is very clear to them, they should be very careful not to imply that causal links are demonstrated by these analytical techniques. It is only suggested, assuming that there is no hidden latent unmeasured variable. Although the authors discuss this limitation later in the manuscript (in the limitation section). I think that they should not let people think that the causal link is there. Epskamp, S., & Fried, E. I. (2018). A tutorial on regularized partial correlation networks. Psychological methods, 23(4), 617. | We agree with the reviewer on this issue, and therefore added an additional caveat at this point: “Partial correlation networks are exploratory hypothesis-generating structures that are merely indicative of potential causal effects []. With this caveat in mind, ….” |
| Comments of Reviewer #3 | Author’s reply |
| The authors replied that the purpose of the pilot experiment was not to achieve iso-intensity. I would agree that this would have been problematic in light of the objective of modelling. For the current objective, it is ideal for the odorants to cover a range of intensities so that there is variation. The authors state that extreme differences in intensity are undesirable and seem to aim for intensities to comparable (page 10). This leaves me in the dark as to the range of intensities they would deem acceptable or comparable. It now appears as if they “just went” with whatever the concentration was that the supplier sent them. Usually, dilutions would be made from those (in general high-intense) odorants the supplier will have prepared, to finetune the range of concentrations to cover a pre-agreed range of intensities. I understand that with Sniffin’ Sticks already prepared by Burghart (with the odorant already in the Stick) it defies the purpose to have to go back to ordering the chemical and prepare the stick all over again, although these were prepared to be comparable and not vary too much. For odorants ordered from suppliers dilutions to achieve variation could have been made.  The authors have addressed the variation for valence, which clearly differed. Can the authors now address address the variation in intensity and if not by providing data from their pilot, by reflecting on this issue? For an example of how concentrations determination is done to match intensities please see Keller, A., Hempstead, M., Gomez, I.A. et al. An olfactory demography of a diverse metropolitan population. BMC Neurosci 13, 122 (2012). https://doi.org/10.1186/1471-2202-13-122 for determination of “high” and “low” levels. I would agree would be exaggerated to put this much work into determination of range for the present paper, but compared with this example I think the authors can do more than they currently do to address this. The means vary between 43 and 77 which strikes me as a bit narrow, but you can convince me otherwise.  Why not insert the table of means in the main paper? | We now report the means and standard deviations of valence, arousal, familiarity and intensity in Table 1.  We now report the range we aimed for in  Section 2.2.: “To ensure the compatibility between the samples, we adopted the criterion set by the developers of the Sniffin’ Sticks that all intensities should be within about 25% of the mean intensity [116].”  And we verified that we succeeded in obtaining this range in Section 3 results:  “The mean intensity ratings listed in Table 1 show that the set of odor stimuli contained no outliers in intensity: all intensities were within about 28% of the mean intensity (which closely agrees with the criterion of 25% set by the developers of the Sniffin’ Sticks [116]). |
| When it comes to safety levels of tar and diesel fume, the authors now refer the reader to find this out for themselves on the supplier website. It is not the responsibility of the reader to verify safety, but that of the scientists. Based on the supplier information can the authors include a declaration in the paper that levels presented were safe? This is not to be dogmatic, but also to state that including such a statement to show that due diligence has been performed in protecting the participant’s health. | We added the following statement about the safety of the samples: “All samples were prepared in compliance with the safety Standards of the International Fragrance Association/” |
| I searched through the manuscript to find the difference baseline affective state and current affective state, but now conclude they are the same. If that is indeed the case, it would help to consistently use the same term. | We now consistently us the term “baseline affective state” throughout the manuscript. |
